# Diversity and antagonistic potential of marine microbes collected from south-west coast of India

**DOI:** 10.1007/s13205-015-0318-1

**Published:** 2015-12-31

**Authors:** S. Sinimol, A. R. Sarika, A. Jayakumaran Nair

**Affiliations:** 1 Department of Biotechnology, University of Kerala, Kariavattom, Thiruvananthapuram, Kerala 695581 India; 2 Kerala State Council for Science, Technology and Environment, Sasthra Bhavan, Pattom, Thiruvananthapuram, Kerala 695004 India

**Keywords:** Microbial diversity, Marine microbes, Bioactivity, *Bacillus* sp. BCS4

## Abstract

The diversity of some of the culturable microorganisms associated with marine flora and fauna collected off Vizhinjam and Mulloor coast of South India was evaluated and their bioactive production potential determined. From a total of 24 bacteria, 4 actinomycetes and 8 fungi isolated from diverse marine sources, five bacterial species-BLM3, BSP2, BCS1, BCS4 and BMA6 showed inhibitory activity against at least one of the tested pathogens viz., *Klebsiella pneumonia* KU1, *Pseudomonas aeruginosa* VL3, *Salmonella enterica typhimurium* MTCC 98, *Escherichia coli* MTCC 40, *Micrococcus luteu*s MTCC 105, *Staphylococcus simulans* MTCC 3610, *Proteus vulgaris* MTCC 426, *Vibrio fluvialis, Vibrio* sp. P3a *and Vibrio* sp. P3b. The isolated actinomycetes and fungi did not produce significant inhibition zones against the tested pathogens; however, the macroalgal isolated actinomycetes strain AMA1 produced reddish pigment in Starch Casein medium which remained stable till the stationary phase of growth. The marine sediment isolate BCS4, identified as *Bacillus* sp. showed wide spectrum of activity against the tested Gram positive bacteria, *S. simulans* MTCC 3610 and Gram negative bacteria, *Proteus vulgaris* with zone of inhibitions of 25 and 11 mm respectively. Better extraction of the bioactive compound was obtained with ethyl acetate when compared with methanol, benzene and hexane and TLC analysis revealed the presence of an active compound. The 16SrRNA sequencing confirmed the potent strain belong to *Bacillus* sp. and hence designated *Bacillus* sp. BCS4.

**Electronic supplementary material:**

The online version of this article (doi:10.1007/s13205-015-0318-1) contains supplementary material, which is available to authorized users.

## Introduction

The increasing antibiotic resistance pose serious concerns in health sector and necessitates seeking natural alternatives. The marine ecosystem being less explored, have prospects for finding novel bioactive producing microbes. Marine microbes represent a distinctive group of organism owing to their immense genetic (Strobel and Daisy 2003) and biochemical diversity (Rusch et al. 2007) and are rich sources of a large variety of bioactive compounds (Debbab et al. 2010). These originate mainly in sediments but they are also present in open oceans in association with other marine organisms (Supriya and Yogesh 2010). Marine invertebrates and plants, in particular, represent an environment rich in microorganisms that produce compounds with bioactive properties including antibacterial, antifungal, antiviral, anticancer, antifouling and antibiofilm activities (Glöckner et al. 2012). The microbial symbionts were been crucial in the discovery of many bioactive compounds reported earlier. The competition among microbes for space and nutrients is one of the driving forces behind the production of precious antibiotics and other useful pharmaceuticals in the marine environment (Thakur et al. 2005b). Microorganisms associated with marine invertebrates are proved to be valuable candidates for drug discovery (Jensen and Fenical 2000; Hentschel et al. 2003; Imada 2004; Thakur et al. 2005a).

The marine microbes include cellular life forms such as bacteria, fungi and plankton, along with viruses that free load along with them. The marine bacteria represent one of the hardly untapped sources of potent therapeutic and novel drug leads. The emergence of multidrug resistant bacteria poses a major threat which in turn forces the discovery of new potent drugs to replace the conventional antibiotics. The decline in the discovery of newer antibiotics of the terrestrial origin necessitates exploring new ecological niches most notably the marine. Marine bacteria showing antibacterial activities have been isolated from various biotypes and most frequently isolated strains belong to the genus *Bacillus, Micrococcus, Pseudomonas, Vibrio, Flavobacterium,* and *Alteromonas* (Jayanth et al. 2001; Nithya et al. 2011; Eltmany et al. 2014; Marinho et al. 2009; Baranova and Eqorov 1989; Leon et al. 2010). However, a major constraint in the discovery of novel drugs from the marine microbes rely up on the fact that majority of the marine microbes are uncultivable in the laboratory conditions. Nevertheless, using cultivated microorganisms is still the only way to get detailed information about microbial characteristics and processes, thus highlighting the need to further focus on culturing microorganisms and developing better culturing techniques (Glöckner et al. 2012).

Marine microbes are rich and yet less harnessed source of structurally diverse secondary metabolites, many of which possess unique biological activities. The present study attempts to isolate and screen novel bioactive producing microbes from the Vizhinjam and Mullor area of the South-west coast of India, which are known for their rich biodiversity and novel compounds in them.

## Materials and methods

### Collection of samples

The marine samples for the isolation of microorganisms were collected from the Vizhinjam and Mulloor coast of Kerala, India. The geographical position of Vizhinjam coast is Longitude E 76°59′ and Latitude N 8°22′.

The samples collected include the Sepia and Crab from the fish landing centre in Vizhinjam coast and Limpets, macroalgae and the sediments from the Mulloor coast. The samples except marine sediments soon after collection were washed with sterile distilled water to remove the soil and brought to the laboratory in sterile polythene bags.

### Isolation of associated microbes from the marine samples

The samples viz., crab, sepia and limpets after removing their outer shell and intestine and the macroalgae were crushed. The resultant was serially diluted in normal saline (0.85 % NaCl) and 0.1 ml spread plated in Zobell Marine Agar (Hi-Media), glucose agar and starch-casein agar to determine the growth of bacteria, fungi and actinomycetes respectively. Similarly, the coastal sediments were serially diluted and plated. The plates were monitored for 48 h by incubating at 37 °C for distinct bacterial colonies. The plated glucose agar was incubated at 27 ± 2 °C for 48 h and starch-casein agar at 27 ± 2 °C for 5–7 days to examine the growth of marine fungi and actinomycetes respectively. In all the cases, CFU/ml was recorded and the morphologically distinct colonies of the bacteria, fungi and actinomycetes were streak plated in respective agar plates to get the auxenic microbial isolate. The pure cultures of the isolates were stored in glycerol stocks at refrigeration temperatures for further studies.

### Screening of antibacterial activity of the isolated marine microbes

The indicator strains used to determine the antibacterial activity of the isolated marine bacteria, fungi and actinomycetes included both the human pathogenic and fish pathogenic bacteria and are listed in Table 1. The bacterial cultures were stored in agar slants at refrigeration temperatures and were activated in fresh nutrient agar medium as and when required.

**Table 1:** Indicator bacterial strains used for screening the antibacterial activity of isolated Marine microorganisms

Sl. no.	Bacterial strains	Source
1	*Klebsiella pneumoniae* KU1	Clinical isolate
2	*Pseudomonas aeruginosa* VL3	Clinical isolate
3	*Salmonella enterica typhimurium* MTCC 98	MTCC^a^
4	*Escherichia coli* MTCC 40	MTCC
5	*Micrococcus luteu*s MTCC 105	MTCC
6	*Staphylococcus simulans* MTCC 3610	MTCC
7	*Proteus vulgaris* MTCC 426	MTCC
8	*Vibrio fluvalis*	Fish pathogen
9	*Vibrio* sp. P3a	Marine isolate
10	*Vibrio* sp. P3b	Marine isolate

^a^Microbial type culture collection

The isolated marine bacteria were inoculated on to 10 ml of Zobell Marine Broth and incubated under shaking conditions at 37 °C for 48 h. After the respective incubation period, 1 ml of sample was withdrawn from the culture flask and centrifuged at 10,000 rpm for 15 min. The pellet was discarded and the supernatant so obtained was filtered using 0.22 µm microfilter (Hi-Media). The resultant cell free supernatant was examined for antibacterial activity against the indicator bacterial strains. For determining the antibacterial activity of the fungal isolates and actinomycetes, the strains were inoculated in glucose broth and starch-casein broth and incubated for 5 and 7 days respectively under shaking conditions. The indicator strains were activated in Nutrient Broth (Hi-Media) by incubating the inoculated broth for 24 h at 37 °C. The antibacterial activity was detected using the well-diffusion method (BSI 1968). The bacterial culture was swabbed on to Mueller–Hinton agar (Hi-Media) plates. Using a cork borer, wells of 7 mm diameter was made on the agar plates containing the lawn of indicator bacterial strain. The cell free supernatant (50 µl) of the marine microbes was added to each well and incubated for 24 h at 37 °C. The plates were observed for the zones of inhibition around the well and the zone diameter (mm) was recorded.

### Determination of growth characteristics of the potent bacterial isolate

Among the microbes screened for bioactive production, active marine bacterial isolate BCS4 was subjected to determination of growth characteristics for effective extraction of the bioactive compound. The bacterial isolate BCS4 was inoculated in 100 ml Zobell Marine Broth and growth and bioactivity was determined every 24 h interval for 120 h. The determination of growth was carried out by taking the optical density of the inoculated culture broth at 600 nm after every 24 h. The bioactivity was determined by assaying the cell-free supernatant against the indicator strains selected by well-diffusion method as described earlier.

### Extraction and chemical screening of bioactive compound from the potent isolate

The potent isolate was inoculated into 100 ml of Zobell Marine Broth and incubated at 37 °C for 96 h. The culture was then centrifuged at 10,000 rpm for 15 min and the supernatant filtered. The resultant filtrate was extracted separately with solvents viz., methanol, ethyl acetate, benzene and hexane @ 1 ml/ml culture supernatant of the marine isolate and dried at room temperature (28 ± 2 °C); in order to determine the best solvent extraction strategy for this bioactive compound. The well-diffusion assay method was used to determine the bioactivity of the extracted compounds. The solvent which extracted the compound better was used for the further extraction of the bioactive compound.

Thin layer chromatography (TLC) technique was employed to screen the bioactive compound. The crude ethyl acetate extract obtained from *Bacillus* sp. BCS4 was partially purified by TLC using silica gel coated chromatography plates. In order to determine the best solvent system for effective separation of crude compound, solvents such as ethyl acetate, methanol, chloroform and water were used in the proportions viz., (1) Ethyl acetate:Methanol:Water (20:2.7:2; v/v/v), (2) Ethyl acetate:Methanol:Water (20:2.8:1; v/v/v) and (3) Chloroform:Methanol (9:1; v/v). An aliquot of crude extract was spotted onto the silica gel plate and allowed to dry for a few minutes. Afterwards, the plate was developed with the solvent as mobile phase in the earlier mentioned proportion in a previously saturated glass chamber with eluting solvents for 30 min at room temperature. The developed plate was dried under normal air and the spots were visualized under visible light. Retention factor (Rf) value of the spot separated on the TLC Plate was determined by adopting the formula,

$${\text{Rf value}} = \frac{\text{Movement of solute from the origin}}{\text{Movement of the solvent from the origin}}$$

The TLC plates were observed for distinct bands. The compound was eluted from the developed plate by scrapping off silica gel and mixed well with ethyl acetate and centrifuged for 10 min at 10,000 rpm. The supernatant was subjected to determination of bioactivity using well-diffusion assay against the indicator strain *S. simulans* MTCC3610 and observed for inhibition zone after incubation at 37 °C for 24 h.

### Molecular characterization of the strain BCS4

The molecular characterization of the active marine bacterial isolate which showed maximum inhibitory activity against the indicator strains tested were subjected to biochemical and molecular characterization studies based on 16S ribosomal RNA sequencing. The total genomic DNA extraction of the active bacteria cultured in Zobell Marine Broth was carried out by Phenol/chloroform extraction method. The 16S rRNA gene of the isolate was amplified using Universal Eubacterial 16S rRNA gene primers and sequenced. The resultant 16S rRNA gene sequence from the potent isolate BCS4 was compared with other bacterial sequences from the GenBank nucleotide database with BLAST (Altschul et al. 1990) to analyse pairwise homology and phylogenetically analyzed using MEGA 6.01 software.

## Results

### Isolation of microorganisms from the marine invertebrates, macroalgae and coastal sediments

**Fig. 1:**
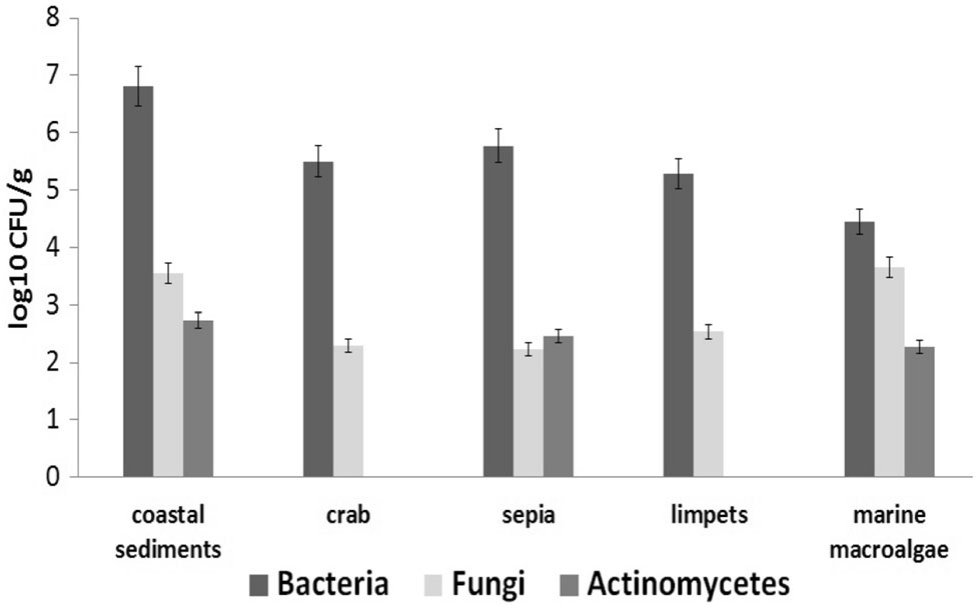
Marine microbes associated with samples collected off south-west Coast of India

The samples from the marine environment (Vizhinjam and Mulloor Coast) used for the isolation of marine bacteria, fungi and actinomycetes include the coastal sediments, marine invertebrates viz., crab, sepia and limpet and the marine macroalgae. The isolated microorganisms from the respective agar plates after spread plating and incubation for 24 h were streaked to get pure cultures of the same. The bacteria were grown in Zobell Marine Agar medium; the list and source of the isolated bacterial strains are presented in Table 1. The total count of bacteria per ml of the sample viz., coastal sediments, crab, sepia, limpets and the marine macroalgae was 6.5 × 10^6^, 3.2 × 10^5^, 5.9 × 10^5^, 1.9 × 10^5^ and 2.8 × 10^4^ CFU respectively (Fig. 1).

The actinomycetes isolated from different samples in starch-casein agar plates after spread plating and incubation for 7 days were streaked onto fresh starch-casein agar plates for getting pure culture of the same. The isolated actinomycetes from different marine sources are given in Table 2. The macroalgal isolate AMA2 produced a pigmented colony upon incubation which yielded a red coloured pigmentation in the starch casein agar plate as well in the culture broth up on 7 days incubation. The total count of actinomycetes per ml of the sample viz., coastal sediments, crab, sepia, limpets and the marine macroalgae was 5.5 × 10^2^, 0, 2.9 × 10^2^, 0 and 1.9 × 10^2^ CFU respectively (Fig. 1).

**Table 2:** The bacterial, fungal and actinomycetes strains isolated from marine samples

Marine sample	Microbes isolated		
	Bacteria	Fungi	Actinomycetes
Coastal sediments	BCS1	FCS1	ACS1
	BCS2	FCS2	ACS2
	BCS3		
	BCS4		
Crab	BCB1	FCB1	–
	BCB2	FCB2	
Sepia	BSP1	FSP1	ASP1
	BSP2	FSP2	ASP2
	BSP3	FSP3	
	BSP4	FSP4	
	BSP5		
Limpets	BLM1	FLM1	–
	BLM2	FLM2	
	BLM3		
Macroalgae	BMA1	FMA1	AMA1
	BMA2	FMA2	AMA2
	BMA3		
	BMA4		
	BMA5		
	BMA6		
	BMA7		
	BMA8		
	BMA9		

### Screening of antibacterial activity of the isolated marine microorganisms

Of the 23 bacteria isolated from different marine sources, only five (22 %) bacterial strains viz, BLM3, BSP2, BCS1, BCS4 and BMA6 showed inhibitory activity against at least one of the tested strains when detected by well-diffusion method. The results revealed that the isolate from coastal sediments (BCS4) exhibited a broad activity spectrum inhibiting both Gram positive (*M. luteus* and *S. simulans*) and Gram negative (*Proteus vulgaris* and two *Vibrio* sp. P3a and P3b) bacteria.

The spectrum of inhibitory activity was narrow for the four strains (BLM3, BSP2, BCS1 and BMA6) which inhibited either one of the indicator strains. The coastal sediment isolates BSP1 and BSP4 exhibited 100 % inhibition against the marine *Vibrio* sp. P3a with zone of inhibition of 25 and 31 mm respectively. None of the marine actinomycetes and fungal isolates showed inhibitory activity against the tested pathogens. Hence, the bacterial isolate BCS4 which showed the highest bioactivity was subjected to further molecular characterization and elution of antibacterial compound.

**Fig. 2:**
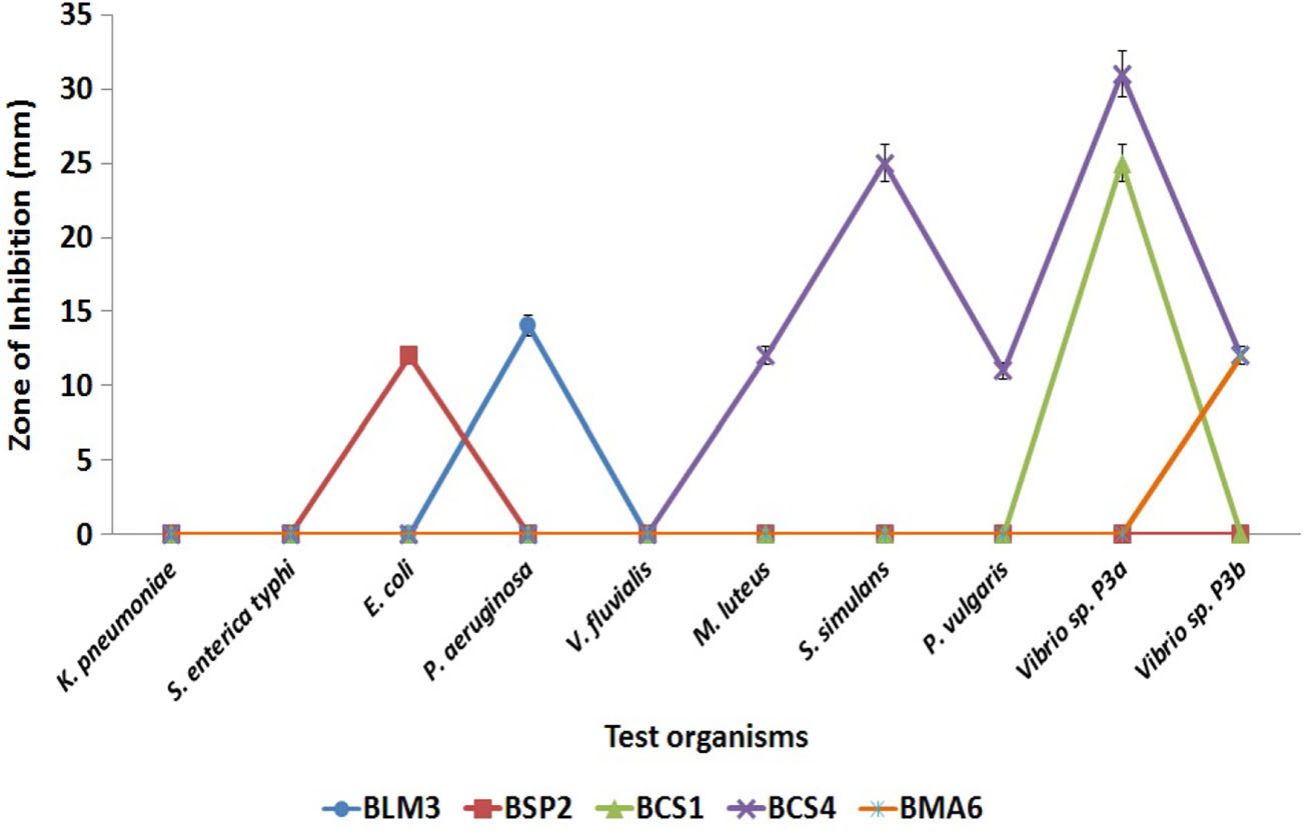
Antibacterial activity of Marine bacterial isolates

The isolated actinomycetes and fungi did not produce significant inhibition zones against the tested pathogens; however, the macroalgal isolated actinomycetes strain AMA1 produced reddish pigment in Starch Casein medium which remained stable till the stationary phase of growth (Fig. 2).

### Growth characteristics and bioactive compound production by the bacterial isolate BCS4

**Fig. 3:**
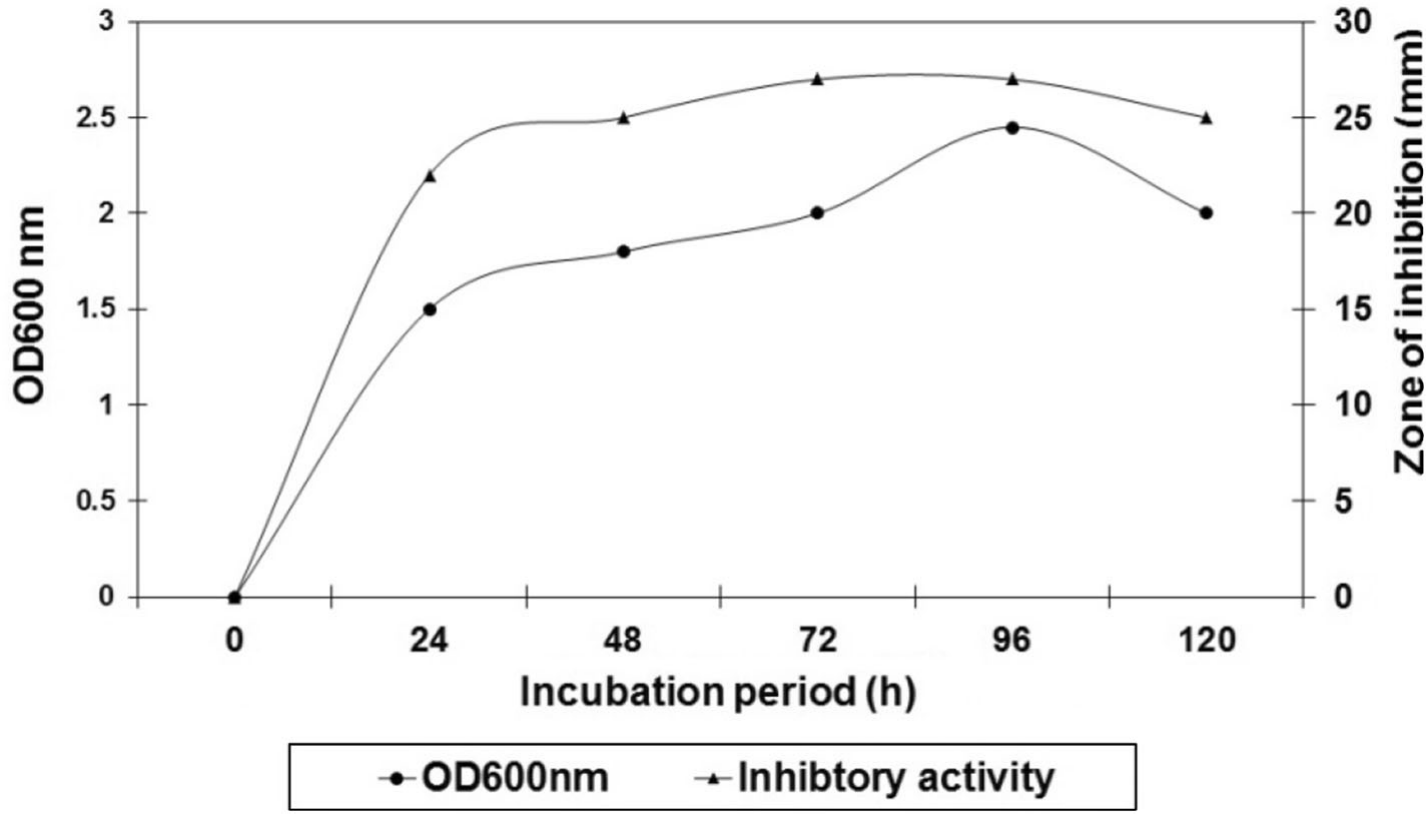
Growth characteristics andbioactive compound production of Marine bacterial isolate BCS4

The determination of growth of the potent marine isolate *Bacillus* sp. BCS4 revealed that the growth initiated after about 8 h of incubation, entered into the exponential phase after 24 h. The bacterium showed a steady growth till the 96th h after which it ceased to grow and entered the stationary phase followed by the decline. The steady rise in the growth rate indicated that the production of the metabolites maximized around 48 to 72 h (Fig. 3). The growth was followed by the production of bioactive metabolite which maximized at the 72th h of incubation and remained stable till the 96th h and decreased thereafter.

### Solvent extraction of the bioactive compound

**Fig. 4:**
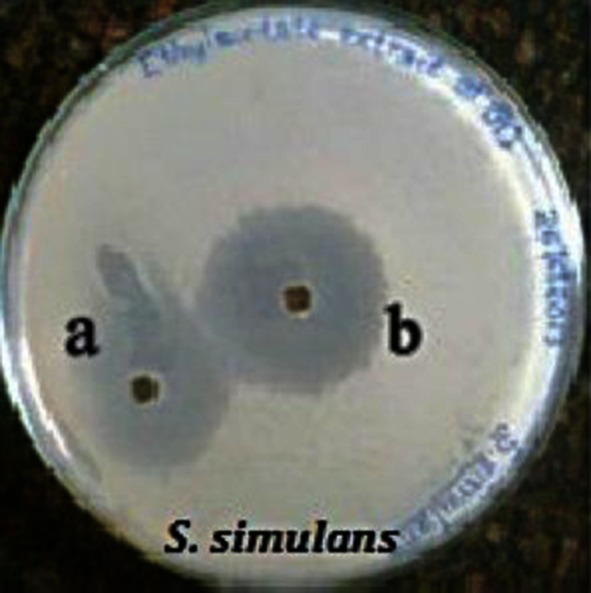
Zone of antibacterial inhibition of ethyl extract of *Bacillus* sp. BCS4 against *S. simulans* MTCC3610. **a** Ethyl acetate extract, **b** compound eluted from silica gel plate

Among the solvents tested for better extraction of the bioactive compound, ethyl acetate extract gave wide zone of inhibition (Table 3) against the test strains indicating the superiority of ethyl acetate over other solvents in extracting the bioactive compound of BCS4 (Fig. 4).

**Table 3:** Activity based extraction of the bioactive compound from *Bacillus* sp. BCS4 using different solvents

Solvents	Zone diameter (mm)	
	*S. simulans*	*P. vulgaris*
Methanol + BCS4	17.8 ± 0.3	6.67 ± 0.6
Benzene + BCS4	13.7 ± 0.6	6.8 ± 0.3
Ethyl acetate + BCS4	27.0 ± 0.3	11.7 ± 6
Hexane + BCS4	–	–
Methanol alone	–	–
Benzene alone	–	–
Ethyl acetate alone	–	–
Hexane alone	–	–

Well diameter = 5 mm; ‘–’ indicates absence of inhibition zone

The partial purification and chemical screening of the bioactive compound from the crude ethyl acetate extract was carried out using Thin Layer Chromatography. Distinct band was detected irrespective of the solvent systems, which is indicative of the presence of a compound in the crude extract. The Rf value was measured as 0.60, 0.72 and 0.79 cm for the solvent systems 1, 2 and 3 respectively. The ethyl acetate extracted compound eluted from the silica gel plate gave a zone of inhibition of 27.2 ± 0.3 mm against *S. simulans* MTCC 3610 confirming the bioactive potential.

### Molecular characterization of the active marine bacterial isolate

**Fig. 5:**
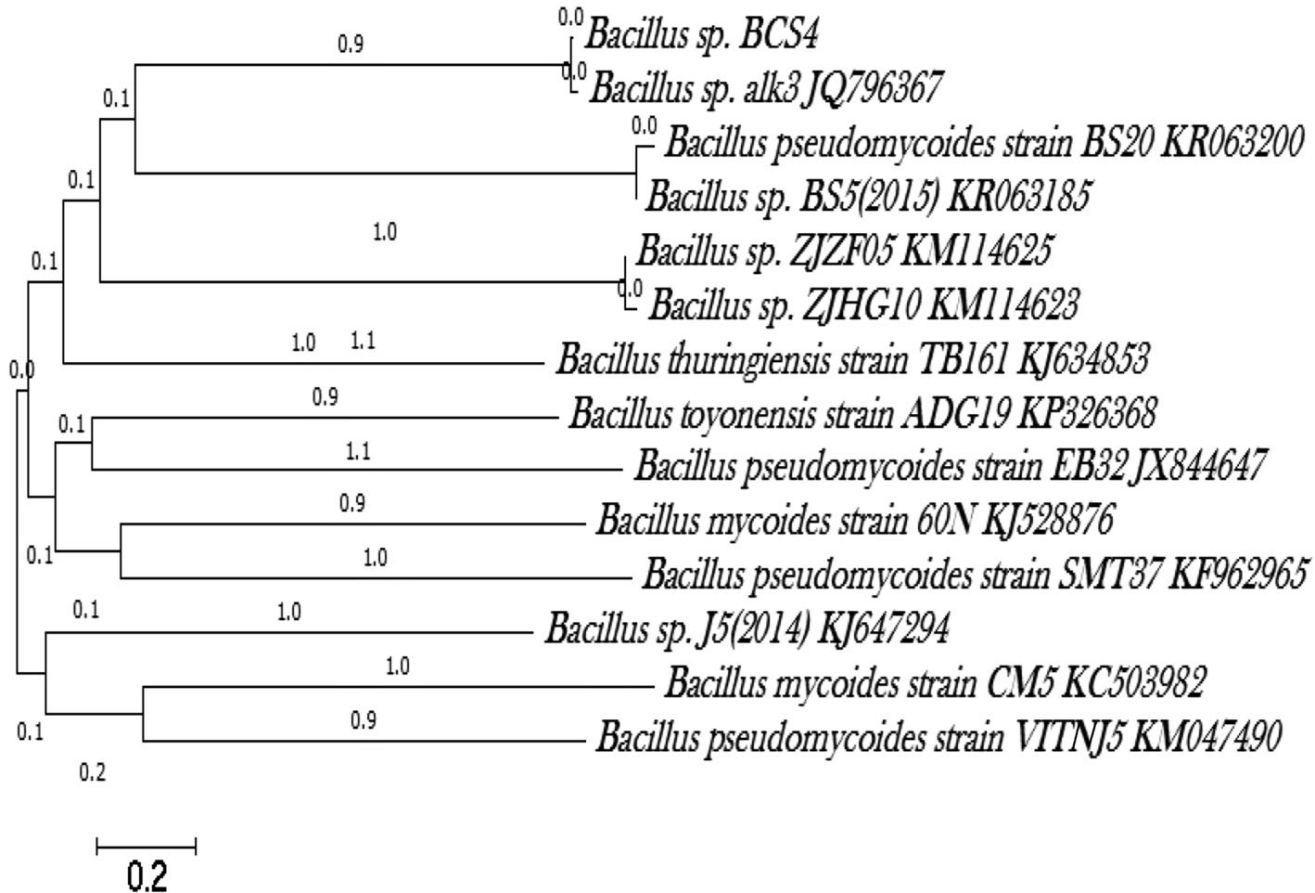
Phylogenetic tree drawn in MEGA 6.0 software using Neighbour Joining Method

The bacterial strain BCS4 which inhibited majority of the tested pathogens was characterized using 16S rRNA gene sequencing and the obtained sequence (749 bp) was blasted using Megablast tool of GenBank (http://www.ncbi.nlm.nih.gov). The blast results revealed 98 % similarity with the *Bacillus* sp. alk3. The phylogenetic tree was constructed through Neighbour Joining Method using Mega 6.0 software (Fig. 5). The active marine isolate was characterized as *Bacillus* sp. and designated *Bacillus* sp. BCS4.

## Discussion

The marine ecosystem is less harnessed in terms of developing alternative drugs to antibiotics. The rich diversity, along with extreme physical conditions makes marine environment an ideal source for proving novel drug leads. The coasts, Vizhinjam and Mulloor along the south-west coast of India harbour rich and biodiverse flora and fauna with millions of microorganisms associated with it. When compared to the terrestrial counterparts, these flora and fauna and the marine environment, in general possess novel drugs and other chemically diverse bioactives (Delong 2007). Though the percentage of culturable microbes remains too little, these can provide insights into the characteristics and potency of microbes (Glöckner et al. 2012) thriving in such extreme conditions.

The marine microbial biota is rich and diverse. The study on the diversity of microbes from coastal sediments and marine flora and fauna revealed that the percentage of bacteria isolated from the studied marine sources was comparatively higher than actinomycetes and fungi. While no actinomycetes were isolated from limpets and crab, at least two fungal strains were isolated from them. Similar observation was made by Tawiah et al. (2012) for the samples collected from Cape Coast Duakor Sea beach at Gulf of Guinea in which the frequency of bioactive producing bacteria was high compared to actinomycetes and fungi. The results from that study also revealed 27 % antibiotic producing microorganism out of 119 isolated recovered. These observations coincided with the observations of the present study which gave 22 % bioactive producing bacteria. On the contrary, high rate of isolation of antibiotic producers (70 %) was recorded by O’Brien et al. (2004) from the Amazon basin.

The highest number of active isolates was obtained from the coastal sediments collected from Vizhinjam coast. The increased number of active isolates in the sediments than from the marine flora and fauna studied might be attributed to fact that the area of sample collection being a Fish Landing area and prone to pollution with fish wastes and other waste discharges, the microbes there might have accustomed to thrive by producing defense compounds. The findings are supported by the observations of Tawiah et al. (2012). The study also revealed active compound producing bacteria and the absence of inhibitory potential by isolated Actinomycetes and Fungi. The absence of the zone of inhibition by the fungal and actinomycetes strains against the tested pathogens might be attributed to the changes in culture conditions or the limitations in the strains tested etc. Further, the actinomycetes and fungi require condition optimization for the production of the bioactive compounds. This would require the standardization of the culture conditions or supplementation with different media components to enrich the medium to enable the production of such compounds. The study carried out Kiranmayi et al. (2011) employed 11 different culture media in order to determine the best production media for the isolated actinomycetes. The variations noted in the antibiotic producers among the isolated strains may also be due to it’s dependence on the isolation and assay procedure, test organisms, type of media used and the sources of bacterial isolates (Giudice et al. 2007). A salient finding noted from the present study that the actinomycete strain AMA1 produced a reddish pigment in Starch-Casein medium which remained stable till the stationary phase of growth. Earlier reports evidenced the production of antitumour (Soliev et al. 2011; Olano et al. 2009), antibacterial (Pham et al. 2014) and other commercially important pigments from marine Actinomycetes. Studies also revealed prognosin like pigment production by Actinomycetes of marine origin (Quadri and Asgar 2012; Chaudhary et al. 2013). The studies on production and determination of the nature of the pigment produced by the strain AMA1 are needed further for characterization and evaluating it’s applicational potentiality.

The bacterial isolate from coastal sediments (BCS4) exhibited a broad activity spectrum inhibiting both Gram positive (*Micrococcus luteus* and *Staphylococcus simulans*) and Gram negative (*Proteus vulgaris* and two *Vibrio* sp. P3a and P3b) bacteria. Similar broad activity spectrum was observed with marine *Pseudoalteromonas* species as was observed by Isnansetyo and Kamei (2003). The activity spectrum observed with the marine isolate in the study provides prospects for a future drug or bioactive lead. The growth pattern of the bacterial isolate revealed that the active metabolite production maximized at the late logarithmic phase (i.e., at the 72th h of incubation) and remained stable till the 96th h. Similar to this observation, Radhakrishna et al. (2011) noted maximum active metabolite production of sponge isolated *Bacillus subtilis* MTCC 10619 at the 72th h, which decreased thereafter.

The isolate with potent activity was phylogenetically confirmed as *Bacillus* sp. BCS4. It is evidenced from the reports of Mondol et al. (2013) that marine Bacillus isolates produce diverse bioactive metabolites with novel modes of action and has potentials for the development of effective management strategies to combat human, animal and phytopathogens in biorational manners. The activity spectrum noted for *Bacillus* sp. BCS4 is indicative of their possible medical and pharmaceutical applications. The compound was effectively extracted using the ethyl acetate solvent and the confirmation of the active compound in TLC provides insights into development of potent drugs out of this compound. Further work to detect the compound chemistry and the purification of the compound to homogeneity is under progress.

## Electronic supplementary material

Below is the link to the electronic supplementary material.

Supplementary material 1 (JPEG 50 kb)

Supplementary material 2 (JPEG 42 kb)

Supplementary material 3 (JPEG 119 kb)

## References

[CR1] Altschul SF, Gish W, Miller W, Myers EW, Lipman DJ (1990). Basic local alignment search tool. J Mol Biol.

[CR2] Baranova NA, Eqorov NS (1989). Production of antibiotic substances by natural variants of the marine bacterium *Vibrio fischeri*. Antibiot Khimoter.

[CR3] BSI (1968) Methods on microbial examination for dairy purposes. British Standards Institution, British Standards House, London, BS. 4285

[CR4] Chaudhary HS, Soni B, Shrivastava AR, Shrivastava S (2013). Diversity and versatility of actinomycetes and its role in antibiotic production. J App Phar Sci.

[CR5] Debbab A, Aly AH, Lin WH, Proksc P (2010). Bioactive compounds from marine bacteria and fungi. Microb Biotechnol.

[CR6] Delong EF (2007). Microbial domains in the ocean: a lesson from the Archae. Oceanography.

[CR7] Eltmany EE, Abdelmohsen UR, Ibrahim AK, Hassanean HA, Hentschel U, Ahmed SA (2014). New antibacterial Xanthone from the marine sponge derived *Micrococcus* sp. EG45. Bioorg Med Chem Lett.

[CR8] Giudice AL, Bruni V, Michaud L (2007). Characterization of Antarctic psychrotrophic bacteria with antibacterial activities against terrestrial microorganisms. J Basic Microbiol.

[CR9] Glöckner FO, Stal LJ, Sandaa R-A, Gasol JM, O’Gara F, Hernandez F, Labrenz M, Stoica E, Varela MM, Bordalo A, Pitta P (2012) Marine microbial diversity and its role in ecosystem functioning and environmental change marine board position paper 17. Calewaert JB, McDonough N (eds) Marine Board-ESF, Ostend, Belgium

[CR10] Hentschel U, Fieseler I, Wehrl M, Gernert C, Steinert M, Hacker J, Horn M, Muller WEG (2003). Microbial diversity of marine sponges. Marine molecular biotechnology.

[CR11] Imada C (2004). Enzyme inhibitors of marine microbial origin with pharmaceutical importance. Mar Biotechnol.

[CR12] Isnansetyo A, Kamei Y (2003). *Pseudoalteromonas phenolica* sp. nov., a novel marine bacterium that produces phenolic anti-methicillin-resistant *Staphylococcus aureus* substances. Int J Syst Evol Microbiol.

[CR13] Jayanth K, Jevasekaran G, Shakila RJ (2001). Biocontrol of fish bacterial pathogens by the antagonistic bacteria isolated from the coastal waters of Gulf of Mannar, India. Bull Eur Ass Fish Pathol.

[CR14] Jensen PR, Fenical W, Fusetani N (2000). Marine microorganisms and drug discovery: current status and future potential. Drugs from the sea.

[CR15] Kiranmayi UM, Sudhakar P, Krishna N, Yellamanda B, Vijayalakshmi M (2011). Taxonomic characterization of potential bioactive metabolite producing actinomycetes from mangrove sediments of Coringa. J Pharm Res.

[CR16] Leon J, Liza L, Soto I, Torres M, Orosco A (2010). Marine Bacteria producing antibacterial compounds isolated from intertidal invertebrates. Rev Peru Med Exp Salud Publica.

[CR17] Marinho PR, Moreira AP, Pellegrino FL, Muricy G, Bastos Mdo C, Santos KR, Giambiagi-de Marval M, Laport MS (2009). Marine *Pseudomonas putida*: a potential source of antimicrobial substance against antibiotic resistant bacteria. Mem Inst Oswaldo Cruz.

[CR18] Mondol MAM, Shin HJ, Islam MT (2013). Diversity of secondary metabolites from marine *Bacillus* species: chemistry and biological activity. Mar Drugs.

[CR19] Nithya C, Devi MG, Pandian KS (2011). A novel compound from marine bacterium *Bacillus pumilus* S6-15 inhibits biofilm formation in gram-positive and gram-negative species. Boufouling.

[CR20] O’Brien A, Sharp R, Russel NJ, Roller S (2004). Antarctic Bacteria inhibit growth of food-borne microorganisms at low temperatures. FEMS Microbiol Ecol.

[CR21] Olano C, Mendez C, Salas JA (2009). Antitumour compounds from marine actinomycetes. Mar Drugs.

[CR22] Pham HT, Nguyen NP, Phi TQ, Dang PT, Le HG (2014). The antibacterial and anticancer activity of marine Actinomycete strain HP411 isolated in the Northern Coast of Vietnam. Int J Med Health Biomed Bioeng Pharm Eng.

[CR23] Quadri RS, Asgar D (2012). Detection of melanin producing thermoalkaliphilic *Streptomyces* from limestone quarries of the Deccan traps. World J Sci Technol.

[CR24] Radhakrishna E, Kumar PS, Sujatha P (2011). Optimization of marine sponge isolated bacterium *Bacillus subtilis* (MTCC No. 10619) for the production of antimicrobial metabolites. Asian J Chem.

[CR25] Rusch DB, Halpern AL, Sutton G, Heidelberg KB, Williamson S (2007). The Sorcer 11 Global ocean sampling expedition: north-west Atlantic through Eastern Tropical Pacific. PLoS Biol.

[CR26] Soliev AB, Hosokawa K, Enomoto K (2011). Bioactive pigments from marine bacteria: applications and physiological roles. Evid Based Complementary Alternat Med.

[CR27] Strobel G, Daisy B (2003). Bioprospecting for microbial endophytes and their natural products. Microbiol Mol Rev.

[CR28] Supriya JS, Yogesh SC (2010). Marine: the ultimate source of bioactives and drug metabolites. Int J Res Ayurveda Pharm.

[CR29] Tawiah AA, Gbedema SY, Adu F, Roamah VE, Annan K (2012). Antibiotic producing microorganisms from River Wiwi, Lake Bosomtwe and the Gulf of Guinea at Doakor Sea Beach, Ghana. BMC Microbiol.

[CR30] Thakur AN, Thakur NI, Indap MM, Pandit RA, Datar VV, Muller WEG (2005). Antiangiogenic, antimicrobial and cytotoxic potential of Sponge-associated bacteria. Mar Biotechnol.

[CR31] Thakur NL, Thakur AN, Mueller WEG (2005). Importance of marine natural products in drug discovery. Natural Product Radiance.

