# Supplementary figures and images for: Diversity and antagonistic potential of marine microbes collected from south-west coast of India
Source: 3 Biotech. 2015 Dec 31;6(1):7. doi: 10.1007/s13205-015-0318-1 (PMC4697912; doi:10.1007/s13205-015-0318-1)

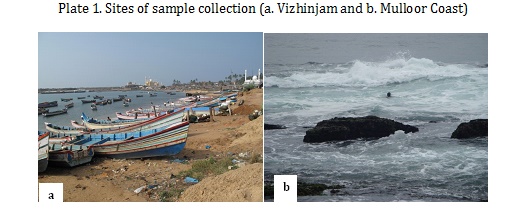

Supplement: Supplementary file 1 — Supplementary material 1 (JPEG 50 kb) [file 13205_2015_318_MOESM1_ESM.jpg]

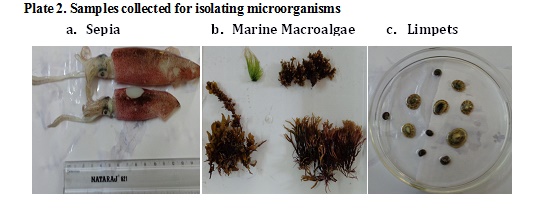

Supplement: Supplementary file 2 — Supplementary material 2 (JPEG 42 kb) [file 13205_2015_318_MOESM2_ESM.jpg]

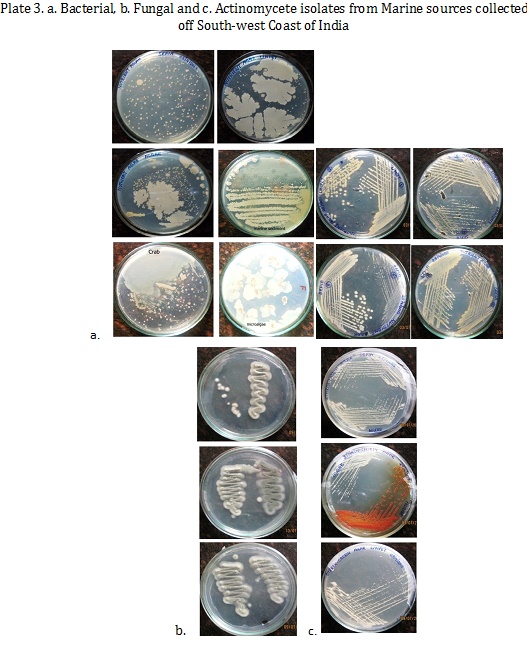

Supplement: Supplementary file 3 — Supplementary material 3 (JPEG 119 kb) [file 13205_2015_318_MOESM3_ESM.jpg]
